# Rheumatoid Arthritis Development following COVID-19 Infection: Eighth Case Described

**DOI:** 10.31138/mjr.151122.rad

**Published:** 2024-02-12

**Authors:** Jozélio Freire de Carvalho, Felipe Freire da Silva

**Affiliations:** Núcleo de Pesquisa em Doenças Crônicas não Transmissíveis (NUPEN), School of Nutrition from the Federal University of Bahia, Salvador, Bahia, Brazil

**Keywords:** rheumatoid arthritis, autoimmunity, COVID-19, coronavirus disease 2019

## Dear Editor,

COVID-19 infection has been associated with the development of several autoimmune diseases.^1^ In addition, anti-citrullinated protein antibodies (ACPA) and rheumatoid arthritis (RA) flaring after SARS-CoV-2 infection have also been described.^2–4^

A 37-year-old previously healthy woman had a history of cough and fever and was diagnosed with COVID-19 infection after positive RT-PCR. She was treated at home with symptomatics (paracetamol and oral hydration) and evolved well after three weeks, with no complications. After three months, she started polyarthritis of her wrists, ankles, and knees, second and third metacarpophalangeal joints bilaterally. She received analgesics and non-steroidal anti-inflammatory drugs with no improvement. She came to our private office; we ordered some tests that showed a positive rheumatoid factor of 68 IU/mL (normal range: < 14 IU/mL), anti-CCP of 200 U/mL (nr: < 20 U/mL), and antinuclear antibodies (1: 320 with a speckled pattern; nr: < 1/80). Her C-reactive protein was 48 mg/L (nr: < 5mg/dL) and erythrocyte sedimentation rate was 23 mm/1st hour (nr: < 20 mm/1st hour). Her x-rays did not show any abnormality. Hand ultrasound showed tenosynovitis of palmar flexor digit and synovitis of second and third metacarpophalangeal and synovitis on her wrists. Infectious serologies were all negative. A diagnosis of rheumatoid arthritis was determined (ACR/EULAR 2010 criteria). The patient denied any family history of RA or other autoimmune diseases. Methotrexate 15mg/week and folic acid 5mg/week were started. She evolved with a marked improvement of her condition after 2 months. After 6 months, she was asymptomatic and inflammatory biomarkers were normal.

This case represents one additional patient who developed RA after a COVID-19 infection.

The case could be a patient with polyarthritis secondary to COVID-19 infection, although all RA-autoantibodies were positive. Moreover, the case fulfilled the international ACR/EULAR 2020 criteria for RA. In addition, other infectious diseases such as hepatitis B, hepatitis C, parvovirus, and Epstein-Barr could cause similar clinical features, although all these infectious serologies were performed in our patient and were negative. Previous seven cases of rheumatoid arthritis following COVID-19 were also reported.^2–6^ In a study by Derksen et al., authors tried to determine the seroprevalence of ACPA after COVID-19. ACPA was measured in 61 patients 5 weeks after hospitalisation; only two patients tested positive for ACPA. These two patients were already previously diagnosed with ACPA-positive RA. Thus, ACPA positivity was not increased after COVID-19^5^ **(Table 1)**. In conclusion, rheumatoid arthritis development after COVID-19 infections has been rarely reported in the literature. In patients with persistent arthritis after COVID-19, RF, and anti-CCP, as well as an image of the joints, should be advised.

**Table 1.** Summary of all published patients with RA following COVID-19.

**Author, year**	**Number, age, sex**	**The time between COVID-19 and RA onset**	**Autoantibody positivity**	**COVID-19 severity**
Derksen et al., 2021^4^	3, ND, ND	6.6 weeks	Anti-CCP	Moderate to severe
Tamborrini et al., 2021^3^	1, ND, ND	ND	RF Anti-CCP	ND
Ben-Chetrit et al, 2021	1, 33, female	12 weeks	Borderline ANA	Mild
Bouzid et al., 2022	1, 38, female	4 weeks	RF 62.9 IU/mL Anti-CCP 237.4 U ANA 1:640	Mild
Drosos et al. 2022	1, 46, female	8 weeks	Negative RF, anti-CCP and ANA	Mild
de Carvalho et al., 2022 (present case)	1, 37, female	12 weeks	RF 68 IU/mL Anti-CCP 200 UANA 1:320	Mild

ANA: antinuclear antibodies; anti-CCP: anti-cyclic citrullinated peptide antibody; ND: not described; RF: rheumatoid factor

## CONFLICT OF INTEREST

The authors declare no conflict of interest.
